# [Expression of Concern] Dexmedetomidine protects against sepsis-associated encephalopathy through Hsp90/AKT signaling

**DOI:** 10.3892/mmr.2026.13843

**Published:** 2026-03-11

**Authors:** Lijun Yin, Xuejun Chen, Hongbo Ji, Shunli Gao

Mol Med Rep 20: 4731–4740, 2019; DOI: 10.3892/mmr.2019.10718

Following the publication of this paper, it was drawn to the Editor's attention by a concerned reader that western blot data featured in Fig. 4A were strikingly similar to data that appeared subsequently in the journal *Journal of Clinical Science* that was written by different authors. Additionally, the right-hand β-actin band in Fig. 1A was apparently the same as the left-hand β-actin band in Fig. 1B; the right-hand band in the gel for cleaved caspase-3 in Fig. 5A was apparently the same as the left-hand protein band for the Bcl-2 blots in Fig. 5B; and the β-actin bands in Fig. 4A seemed to match with most of the β-actin bands in Fig. 1A.

The authors have been contacted by the Editorial Office to offer an explanation for these apparent anomalies in the presentation of the data in this paper; however, up to this time, no response from them has been forthcoming. Owing to the fact that the Editorial Office has been made aware of potential issues surrounding the scientific integrity of this paper, we are issuing an Expression of Concern to notify readers of these potential problems while the Editorial Office continues to investigate this matter further.

